# Supplementary material for: Volatile Organic Compounds in the Azteca/Cecropia Ant-Plant Symbiosis and the Role of Black Fungi
Source: J Fungi (Basel). 2021 Oct 6;7(10):836. doi: 10.3390/jof7100836 (PMC8539435; doi:10.3390/jof7100836)
Supplement: Supplementary file 1 [file jof-07-00836-s001.zip › jof-1376489-supplementary.pdf]

Mayer et al Supplementary tables

Supplementary Table S1. Collection information on the activity of the *Azteca alfari* colonies, which internode (=domatium) was sampled, the total amount sampled per domatium as well as size, length and volume of the sampled domatium are given. Additionally, sampling time, temperature and humidity during the sampling are listed. Do= domatium, sa = see above.

type	colony activity	plant ID	Do no	amount sampled (L)	do vol (L)	do diam (cm)	do length (cm)	do tot	°C	%rh	plant species	plant height (m)	time
**alarmed**	weak	19_6 i9	i9	0.9	0.08	3.5	8.4	40	28.7	82	*obtusifolia*	3.3	15:45-21:20
	strong	19_7 i10	i10	1.06	0.13	5.5	5.6	> 41	27.8	83	*peltata*	c. 5	15:30-21:28
	strong	19_8 i13	i13	1	0.06	4.4	3.9	> 60	27.8	83	*peltata*	c. 6	15:34 -21:30
	medium	19_21 and 19_22	i10	1	0.02	2.2 and 2.4	4.1	21	28.3	89.2	*peltata*	2.5	11:48 -15:37
**not inhabited**		19_21 & 19_22	i1-i3 each	0.8	0.02	2.3	3.9	sa	27.5	97.5	*peltata*	sa	16:05
		19_7 & 19_8	i2 each	1.2	0.07 & 0.05	4.3 & 3.6	4.8 & 4.9	sa	31.8	71.4	*peltata*	sa	08:10 -11:17

Supplementary Table S2. Volatile compounds detected and identified in this study. In total 211 different belonging to 19 different chemical families were identified.

Compound	CAS No.	ug/m3									FORM
		Control1	Control2	Control4	alarm1	alarm2	alarm3	alarm4	domat3	domat4	
		Ambient air			weak colony	vital *A. alfari* colony			uninhabited domatia		
**Alcohols**											
Ethanol	64-17-5	11,1	7,4	5,7	0,8	1,1	1,2	3,8	1,5	1,6	C2H6O
Isopropyl Alcohol	67-63-0	0,1	0,8		0,1	0,3	0,4		5,7	1,4	C3H8O
1-Propanol	71-23-8		0,4	0,3					0,6	0,4	C3H8O
2-Butanol	78-92-2								0,6		C4H10O
1-Propanol, 2-methyl-	78-83-1	0,6	0,7	1,6	1,2	0,4	0,4	0,6	1,2		C4H10O
1-Butanol	71-36-3	2,0	2,6	4,6	3,7	1,1	1,3	1,8	1,7	1,1	C4H10O
2-Butanol, 3-methyl-	598-75-4							0,4			C5H12O
1,3-Cyclobutanediol, 2,2,4,4-tetramethyl-	3010-96-6	0,2									C8H16O2
Ethanol, 2-(2-methoxyethoxy)-	111-77-3								2,2		C5H12O3
Ethanol, 2-(2-ethoxyethoxy)-	111-90-0								5,6		C6H14O3
1-Hexanol, 2-ethyl-	104-76-7	13,5	7,9	7,0	22,7	9,5	4,9	12,6	3,6	3,8	C8H18O
1-Octanol	111-87-5		0,4					0,5		1,4	C8H18O
**Aldehydes**											
Acetaldehyde	75-07-0	2,7	8,0	13,9	4,5	1,6	0,7	9,7	15,3	11,9	C2H4O
2-Propenal	107-02-8	2,9	2,2	1,6	2,2	2,2	2,6	1,5	2,5	2,3	C3H4O
Propanal	123-38-6	0,3	0,5	0,3	0,1	1,2	2,3	5,4	1,4		C3H6O
Propanal, 2-methyl-	78-84-2								0,6	0,6	C4H8O
Methacrolein	78-85-3	3,6	2,2	2,8	1,8	1,3	1,7	1,7	3,0	1,5	C4H6O
Butanal	123-72-8	1,0	0,4	0,7	0,3	0,1	0,5	0,8	1,0	0,1	C4H8O
Butanal, 3-methyl-	590-86-3									0,5	C5H10O
2-Butenal, 2-methyl-	1115-11-3	1,6	0,2	0,5	1,1	1,0	0,9		0,7		C5H8O
Hexanal	66-25-1				1,8					2,2	C6H12O
2-Butenal, 3-methyl-	107-86-8						0,6				C5H8O
Heptanal	111-71-7	0,6	0,8	0,4	0,8	0,5	0,8	0,8	0,8	1,8	C7H14O
Benzaldehyde	100-52-7	3,6	8,0	3,3	2,1	6,2	9,5	10,8	23,3	4,7	C7H6O
Octanal	124-13-0	4,3		0,9			2,7	3,9	2,6	6,5	C8H16O
Nonanal	124-19-6	3,9	3,9	2,4	2,5	3,9	5,3	4,3	2,7	21,1	C9H18O
Decanal	112-31-2	3,6	3,4	1,7	2,1	2,1	2,5	2,9	2,5	8,9	C10H20O
Undecanal	112-44-7		0,2			0,5			0,1	0,7	C11H22O
Dodecanal	112-54-9	1,1	0,5	2,3	0,6	0,4	0,4	1,0	0,7	4,2	C12H24O
Tridecanal	10486-19-8	1,0							0,1	0,5	C13H26O
**Aliphatic Hydrocarbons**											
Butane	106-97-8	1,4	1,2	0,4	0,4	0,5	0,2		0,3		C4H10
1,3-Butadiene	106-99-0	0,3	0,2			0,5	0,3		0,3		C4H6
1-Butene, 3-methyl-	563-45-1				0,9	6,0	4,9	0,7	1,1		C5H10
Butane, 2-methyl-	78-78-4	0,6	0,9	0,4	0,6	0,5	0,9		1,5	0,6	C5H12
Pentane	109-66-0	0,5	0,6	0,4	1,4	2,2	2,8	1,5	2,5		C5H12
2-Pentene, 4-methyl-, (Z)-	691-38-3				0,5	7,1	4,5		0,7		C6H12
Pentane, 3-methyl-	96-14-0	0,8									C6H14
1-Hexene	592-41-6	1,6	0,2	0,1	1,1	1,4	0,7	0,3	0,5	0,3	C6H12
n-Hexane	110-54-3	2,5	1,0	1,1	7,3	0,8	0,9	1,4	1,2	0,3	C6H14
2-Hexene	592-43-8				0,3						C6H12
Hexane, 3-methyl-	589-34-4	1,7									C7H16
1-Heptene	592-76-7	1,3			1,1	1,0	0,6				C7H14
Heptane	142-82-5	1,5	0,4	0,4	2,4	0,5	0,5	0,9	0,5	0,4	C7H16
(Z)-2-Heptene	6443-92-1	0,4			0,5	0,2					C7H14
1-Pentene, 2,4,4-trimethyl-	107-39-1	0,8	0,2			1,3					C8H16
Pentane, 2,3,4-trimethyl-	565-75-3	0,7	1,7	1,3		0,4	0,4	4,0	1,1		C8H18
Pentane, 2,3,3-trimethyl-	560-21-4	1,0	2,9	2,1		0,8	0,6	6,4	0,7		C8H18
Hexane, 2,2,4-trimethyl-	16747-26-5		0,9	0,8		0,2		1,9			C9H20
Heptane, 3-methylene-	1632-16-2		1,3	0,5							C8H16
Octane	111-65-9	0,8	0,4		71,4	0,7	0,6		0,5	0,4	C8H18
Octane, 4-methyl-	2216-34-4	0,4	0,2				0,1	0,4	0,3		C9H20
Octane, 3-methyl-	2216-33-3	0,6							0,4		C9H20
Nonane	111-84-2	1,2	0,7	0,4	2,5	1,4	0,5		0,9		C9H20
Heptane, 3-methyl-	589-81-1	2,2	1,1	0,3		0,3	1,0	1,0	1,6		C8H18
Octane, 2,5-dimethyl-	15869-89-3	1,4	0,5	0,8	0,3		0,9	0,9	0,7		C10H22
Octane, 2,6-dimethyl-	2051-30-1	1,9		1,5	0,7				3,1		C10H22
Heptane, 3-ethyl-	15869-80-4	4,0		0,4	1,1	1,0	2,0	1,5	1,8		C9H20
Nonane, 4-methyl-	17301-94-9	2,9	1,2	0,8			1,8	2,2			C10H22
Nonane, 2-methyl-	871-83-0	4,4	2,0	1,2	1,9	1,4	2,7	3,4	3,5		C10H22
Nonane, 3-methyl-	5911-04-6	4,4	2,6	1,2	1,8	1,6	2,9	2,9	2,9		C10H22
Decane	124-18-5	4,6	2,2	1,6	2,4	1,8	2,8	3,9	3,7	0,8	C10H22
Nonane, 2,6-dimethyl-	17302-28-2	1,0		0,3					1,1		C11H24
Undecane	1120-21-4	2,5	0,8	0,6	1,0	0,4	0,7	1,0	2,0	0,7	C11H24
Dodecane	112-40-3	4,9	2,3	0,9	3,2	2,5	3,8	1,7	3,1		C12H26
Tridecane	629-50-5	1,2	0,6	0,7	0,7	0,6	0,5	0,4	0,6	0,2	C13H28

Tetradecane	629-59-4	2,2	1,6	0,9	2,0	1,6	2,2	1,5	2,7	1,2	C14H30
Hexadecane	544-76-3	0,8	0,7	1,0	0,6	0,8	0,6	0,8	0,8	2,0	C16H34
Heptadecane	629-78-7	0,4	0,4	0,5		0,9		0,3		1,8	C17H36
∑ 2-Butene (isomers)		2,2	0,4		1,5	1,6			10,0	0,4	C4H8
C4H8		6,7	3,6	2,1	3,5	4,6	2,1	1,3	5,0	1,7	C4H8
C5H8		0,4	0,3	0,4	0,4	2,0	1,6	0,7	1,6		C5H8
∑ unknown		0,8	0,2			0,1	0,6	1,2	0,6	0,5	
**Amines**											
Ethenamine, N-methylene-	38239-27-9			0,3						0,3	C3H5N
**Aromatic Alcohols**											
Phenol	108-95-2	4,0			0,7	0,4	1,8	0,4	3,7	1,1	C6H6O
p-Cresol	106-44-5		0,5						0,2		C7H8O
Phenol, 3-ethyl-5-methyl-	698-71-5							0,3			C9H12O
**Aromatic compounds**											
Benzene	71-43-2	4,6	2,9	2,3	3,0	3,0	2,5	1,3	5,5	1,9	C6H6
Toluene	108-88-3	7,7	4,5	2,8	2,7	2,3	4,3	2,7	5,6	1,1	C7H8
Ethylbenzene	100-41-4	0,4	0,2	0,2	0,2	0,1	0,2	0,1	0,5		C8H10
m,p-Xylene		1,5	1,2	1,0	0,9	0,7	1,0	0,9	2,2	0,5	C8H10
Phenylethyne	536-74-3	0,1	0,2			0,1	0,1				C8H6
o-Xylene	95-47-6	0,3	0,4	0,2	0,2				0,4		C8H10
Styrene	100-42-5	0,8	0,4	0,3	1,1	0,4	0,5	0,4	0,7		C8H8
Benzene, 1,2,4-trimethyl-	95-63-6	0,4	0,3	0,1	0,1	0,2		1,1	0,5		C9H12
p-(1-Propenyl)-toluene	429549		20,9				0,4		1,4		C10H12
Naphthalene	91-20-3	0,2	0,1	0,2	0,1	0,1	0,1	0,2	0,1		C10H8
Naphthalene, 2-methyl-	91-57-6	0,2			0,1		0,1	0,1	0,1		C11H10
**Cyclic Hydrocarbons**											
Cyclopropane, ethyl-	1191-96-4	1,7	1,1	0,7	1,6		1,7	1,1	1,0	0,7	C5H10
Cyclopentane, methyl-	96-37-7		1,2			59,3	1,9	30,3	0,6	1,3	C6H12
Cyclopentene, 1-methyl-	693-89-0					0,7					C6H10
Cyclopentene, 1,2,3-trimethyl-	473-91-6					0,9	1,0		0,6		C8H14
Bicyclo[3.1.0]hexane, 1,5-dimethyl-	142175					1,7	1,5		3,5		C8H14
Cyclohexene, 3,3,5-trimethyl-	503-45-7		0,5	0,4					0,2		C9H16
Cyclohexane, ethyl-	1678-91-7	4,0	7,5	8,8		4,1	2,9	11,8	3,2		C8H16
Cyclohexene, 1,6,6-trimethyl-	69745-49-9		0,3	0,3					0,1		C9H16
Cyclopentanone, 3-methyl-	1757-42-2		0,3		2,0						C6H10O
**Esters**											
Methyl acetate	79-20-9	2,6	3,0	8,6	0,4		0,6	1,2	2,1		C3H6O2
Ethyl Acetate	141-78-6	4,6	0,2	0,3	1,4	0,4	0,8	0,3	0,2		C4H8O2
Methyl propionate	554-12-1								0,4		C4H8O2
Ethyl acrylate	140-88-5	0,2								0,2	C5H8O2
n-Propyl acetate	109-60-4						0,6				C5H10O2
Butyl acetate	123-86-4	0,8	0,5	0,3	0,6	0,4	0,4		0,3	0,5	C6H12O2
Methyl valerate	624-24-8								0,6		C6H12O2
Methyl 2,3-dimethylbutanoate	30540-29-5				1,6	5,0	2,0		0,1		C7H14O2
3-Methylcyclopentyl acetate	24070-70-0	0,7	0,5	0,2	0,4	0,4	1,0			0,6	C8H14O2
Methyl 2-ethylpentanoate	816-16-0				2,2	9,9	5,9	2,1	0,4		C8H16O2
Methyl (2E)-4,4-dimethyl-2-pentenoate	16812-85-4				0,3	0,9	1,4				C8H14O2
Methyl 2,4-dimethylhexanoate	14251-45-7						0,8				C9H18O2
Methyl benzoate	93-58-3							0,1	1,5		C8H8O2
Dibutyl phthalate	84-74-2		25,5	14,3		62,8	34,5	21,9		24,0	C16H22O4
**Ethers**											
1,3-Dioxolane	646-06-0						0,4		0,4		C3H6O2
1,3-Dioxolane, 2-methyl-	497-26-7	0,6			0,5	0,5	2,0				C4H8O2
Ethyl-1-propenyl ether	928-55-2	0,2	0,1	0,1	0,3	0,2	0,3	0,1	0,3	0,2	C5H10O
2-Propanol, 1-methoxy-	107-98-2					0,1		0,4			C4H10O2
1,4-Dioxane	123-91-1	1,1	0,4	0,6	1,5	0,6		0,5	1,8	1,3	C4H8O2
Ethanol, 2-ethoxy-	110-80-5	0,3				0,2	1,5				C4H10O2
2,2'-Bi-1,3-dioxolane	6705-89-1						0,7				C6H10O4
Anisole	100-66-3					0,3	0,3	0,7	0,2		C7H8O
Benzene, 1-methoxy-4-methyl-	104-93-8						0,2		0,7		C8H10O
Thymol methyl ether	1076-56-8				0,6			0,6	0,2		C11H16O
**Furans**											
Furan	110-00-9	0,7	0,1	0,2	0,8	0,9	0,8	0,3	0,6		C4H4O
Furan, 2,3-dihydro-	1191-99-7						0,5				C4H6O
Furan, 2-methyl-	534-22-5					1,1	1,9	1,3	4,0		C5H6O
Tetrahydrofuran	109-99-9	0,4	0,2	0,5			0,3	0,5		0,3	C4H8O
Furan, 2-pentyl-	3777-69-3		0,1			0,3	0,2		0,6		C9H14O
**Halogen-containing compounds**											
Ethane, 1,1,2-trichloro-1,2,2-trifluoro-	76-13-1	0,3		0,1		0,2		0,3	0,1	0,2	C2Cl3F3
Methylene chloride	75-09-2	0,1	0,2	0,1							CH2Cl2
**Heterogroups**											
Hydrogen isocyanate	75-13-8		1,1		2,6						CHNO
Acetamide	60-35-5								0,4	0,6	C2H5NO
1,2-Benzisoxazole	271-95-4								0,7		C7H5NO
Diethyltoluamide	134-62-3	0,5	36,5	0,7		58,1	23,4	2,3	0,1	7,8	C12H17NO
**Ketones**											
Acetone	67-64-1		6,6	6,7				5,5	35,8		C3H6O
Methyl vinyl ketone	78-94-4	1,1	1,1	0,7	0,9	1,0	1,2				C4H6O

2-Butanone	78-93-3								2,4	0,5	C4H8O
2-Pentanone	107-87-9		2,0	2,6	2,7	80,1	180,4	460,0	1,6	28,1	C5H10O
Methyl Isobutyl Ketone	108-10-1		0,2			0,2	0,4	0,9	0,6		C6H12O
3-Penten-2-one	625-33-2					1,4	2,0	6,1			C5H8O
2-Pentanone, 3-methyl-	565-61-7					0,3	0,8	1,5			C6H12O
5-Hexen-2-one	109-49-9					4,4	4,9	12,1		0,5	C6H10O
2-Hexanone	591-78-6		0,2	0,3	0,5	19,5	32,4	63,3	0,2	3,5	C6H12O
2-Hexanone, 4-methyl-	105-42-0					0,5	0,5	1,4			C7H14O
Cyclopentanone, 2-methyl-	1120-72-5				7,2						C6H10O
2-Cyclopenten-1-one	930-30-3						0,3				C5H6O
Acetyl valeryl	96-04-8					0,3	0,4				C7H12O2
5-Hepten-2-one	6714-00-7					69,7	146,1	200,0		3,2	C7H12O
3-Heptanone	106-35-4			0,3							C7H14O
2-Heptanone	110-43-0	1,7	176,5	125,3	136,3	3720,6	4868,2	6969,8	67,7	829,8	C7H14O
4-Cyclopentene-1,3-dione	930-60-9					0,3			0,3		C5H4O2
2-Heptanone, 3-methyl-	2371-19-9						0,1	0,2			C8H16O
3-Hepten-2-one	1119-44-4					0,9	1,9	2,0			C7H12O
2-Heptanone, 6-methyl-	928-68-7							0,2			C8H16O
2-Heptanone, 5-methyl-	18217-12-4				0,5	1,2	1,7	3,7			C8H16O
1-Hepten-3-one	2918-13-0						0,3	0,9			C7H12O
3-Heptanone, 6-methyl-	624-42-0					0,3	1,2				-
5-Hepten-2-one, 6-methyl-	110-93-0	0,5	5,2	3,5	0,4	153,2	209,1	309,1	1,8	12,0	C8H14O
Ethanone, 1-cyclohexyl-	823-76-7				20,0						-
Ethanone, 1-(1-cyclohexen-1-yl)-	932-66-1	0,6			2,1						C8H12O
3,6-Heptanedione	1703-51-1				0,3	0,3	0,7	1,0			C7H12O2
2-Nonanone	821-55-6				0,2	1,2	2,2	3,4			C9H18O
Acetophenone	98-86-2	2,3	1,1	1,2	1,2	1,6	2,2	2,3	23,1	2,1	C8H8O
6-Methyl-3,5-heptadiene-2-one	1604-28-0						0,2	0,2			C8H12O
Ethanone, 1-(4-methylphenyl)-	122-00-9	0,2	6,4	0,1	0,2	0,2			0,2		C9H10O
5,9-Undecadien-2-one, 6,10-dimethyl-, (	3796-70-1	0,6	0,4		0,3	0,3	0,6	0,5	0,5	3,4	C13H22O
Benzophenone	119-61-9	0,1	0,4	0,1		0,2	0,3	0,4	0,3	0,4	C13H10O
unknown		0,3	0,8		0,1	0,7	0,8	0,7	0,3	0,2	C5H10O
**Lactones**											
Butyrolactone	96-48-0	1,1	0,7		1,1	0,2	0,9		1,1	0,8	C4H6O2
2H-Pyran-2-one, tetrahydro-	542-28-9	1,0	0,5	0,2	0,5	0,7	0,4	0,3	0,4	0,4	C5H8O2
1(3H)-Isobenzofuranone	87-41-2				0,1				0,3		C8H6O2
**Nitrogen-containing compounds**											
Acetonitrile	75-05-8	2,8	2,2	3,6	2,8	1,3		4,5	2,8	4,4	C2H3N
Propanenitrile	107-12-0		0,1								C3H5N
Isobutyronitrile	78-82-0			0,2					0,3	0,3	C4H7N
Propane, 2-nitro-	79-46-9								0,6	0,7	C3H7NO2
2-Propanone, hydrazone	5281-20-9			0,2	0,2	0,2		0,2			C3H8N2
Benzonitrile	100-47-0	1,7	0,6	0,7	1,6	1,0	1,9	0,5	7,0	1,6	C7H5N
3-Pyridinecarbonitrile	100-54-9	0,3		0,1				0,3		0,3	C6H4N2
Methenamine	100-97-0	1,6	1,0		3,5	0,8	1,3				C6H12N4
**Organic Acids**											
Acetic acid	64-19-7	54,3	1,1	7,7	13,1	7,9	6,7	14,8	23,9	19,6	C2H4O2
Propanoic acid, 2-methyl-	79-31-2				0,5				1,6		C4H8O2
Butanoic acid	107-92-6								0,7		C4H8O2
Pentanoic acid, 2-methyl-	97-61-0								0,6		C6H12O2
Nonanoic acid	112-05-0	0,3				0,6					C9H18O2
1,2-Benzenedicarboxylic acid	88-99-3	3,8	1,3	0,3	4,4	4,2	1,3	0,3	0,2	0,5	C8H6O4
Octyl octanoate	2306-88-9								1,3		C16H32O2
**Oxygen-containing compounds**											
5-(Tetrahydro-2H-pyran-2-yloxy)pentana	14194-86-6								0,4		C10H18O3
Furfural	98-01-1	0,4		0,2	0,7	0,4	0,4	0,4	0,3	0,5	C5H4O2
Ethanol, 2,2'-oxybis-	111-46-6								3,8		C4H10O3
Methyl salicylate	119-36-8						0,1		0,3		C8H8O3
Ethanol, 2-[2-(2-ethoxyethoxy)ethoxy]-	112-50-5								1,0		C8H18O4
Diisobutyl phthalate	84-69-5		19,0	14,3	3,4	47,8	30,7	17,0		16,8	C16H22O4
**Sulfur-containing compounds**											
Sulfur dioxide	7446-09-5	57,4	343,0	675,2	56,0	35,6	20,6	458,9	401,0	830,1	SO2
Dimethyl sulfide	75-18-3	0,1	0,3								C2H6S
Carbon disulfide	75-15-0	3,0	49,4	73,9	1,8	2,5	2,5	88,0	239,3	52,0	CS2
Thiirane	420-12-2		0,5	1,0				1,4	0,8	0,9	C2H4S
Thiophene	110-02-1		1,2	1,4				1,1	4,4	0,9	C4H4S
Disulfide, dimethyl	624-92-0		0,2						0,1	0,2	C2H6S2
Thiophene, 2-methyl-	554-14-3		0,9	1,0				0,6	1,5	0,3	C5H6S
Thiophene, 3-methyl-	616-44-4		0,4	0,5		0,3		0,6	2,5	0,4	C5H6S
Thiophene, 2-ethyl-	872-55-9								0,4		C6H8S
Thiophene, 3,4-dimethyl-	632-15-5								2,0		C6H8S
Benzothiazole	95-16-9	2,6	1,0	0,4	1,6	1,1	1,3	1,6	80,3	2,4	C7H5NS
**Terpenes**											
α-Pinene	80-56-8	0,3	2,9	0,3	0,3	3,6	2,6	7,7	6,9	0,5	C10H16
Camphene	79-92-5		0,2					0,3	3,0		C10H16
Sabinene	3387-41-5		0,3			4,4	7,0	4,8	12,4	0,8	C10H16
β-Myrcene	123-35-3	0,2	1,6	0,2	0,2	8,3	5,7	1,5	9,0	0,5	C10H16
(-)-β-Pinene	18172-67-3			0,1		0,6		4,5	3,4	1,1	C10H16
α-Phellandrene	99-83-2					4,2	0,4		28,3	0,4	C10H16
3-Carene	13466-78-9	0,2	0,9		0,3	0,2		0,2	0,7		C10H16

Limonene	138-86-3					3,5					$C_{10}H_{16}$
D-Limonene	5989-27-5	2,7	231,2	0,7	1,0	7,4	6,2	2,3	27,3	1,2	$C_{10}H_{16}$
p-Cymene	99-87-6	0,6	93,4	0,2	0,5	17,9	10,4	1,1	55,9	2,0	$C_{10}H_{14}$
β-Phellandrene	555-10-2		0,4			5,5	1,6	0,6	26,1	0,6	$C_{10}H_{16}$
Eucalyptol	470-82-6					1,7	3,8	1,5	7,2	1,1	$C_{10}H_{18}O$
γ-Terpinene	99-85-4		1,2		0,5	0,9	0,5	0,3	4,7		$C_{10}H_{16}$
Terpinolene	586-62-9		5,0			0,5			1,4		$C_{10}H_{16}$
Cyperene	2387-78-2				0,6	0,3		1,4		2,1	$C_{15}H_{24}$
Caryophyllene	87-44-5				9,4						$C_{15}H_{24}$
$C_{10}H_{16}$						1,3			4,5		$C_{10}H_{16}$
$\sum C_{15}H_{24}$			0,1				0,2	1,9	0,8		-
**unknown**											
Uncategorized		31,9	26,6	16,2	53,8	57,3	64,4	109,5	62,2	34,1	
